# Denoising Non-Invasive Electroespinography Signals by Different Cardiac Artifact Removal Algorithms

**DOI:** 10.3390/bios16020082

**Published:** 2026-01-29

**Authors:** Desirée I. Gracia, Eduardo Iáñez, Mario Ortiz, José M. Azorín

**Affiliations:** 1 Brain-Machine Interface Systems Lab, Miguel Hernández University of Elche, 03202 Elche, Spain; dgracia@umh.es (D.I.G.); mortiz@umh.es (M.O.); jm.azorin@umh.es (J.M.A.); 2 Engineering Research Institute of Elche—I3E, Miguel Hernández University of Elche, 03202 Elche, Spain; 3 Valencian Graduated School and Research Network of Artificial Intelligence—ValGRAI, 46022 Valencia, Spain

**Keywords:** electrospinography (ESG), electrocardiographic (ECG) artifact, denoising algorithm, adaptive template subtraction (ATS)

## Abstract

The non-invasive recording of spinal cord neuronal activity, also known as electrospinography (ESG), using high-density surface electromyography (HD-sEMG) is a promising emerging biosensing modality. However, these recordings often contain electrocardiographic (ECG) artifacts that must be removed for accurate analysis. Given the emerging nature of ESG and the lack of dedicated signal processing methods, this study assesses the performance of seven established EMG denoising algorithms for their ability to preserve the broad spectral bandwidth needed for future ESG characterization: Template Subtraction (TS), Adaptive Template Subtraction (ATS), High-Pass Filtering at 200 Hz (HP200), ATS combined with HP200, Second-Order Extended Kalman Smoother (EKS2), Stationary Wavelet Transform (SWT), and Empirical Mode Decomposition (EMD). Performance was quantified using six metrics: Relative Error (RE), Signal-to-Noise Ratio (SNR), Cross-Correlation (CC), Spectral Distortion (SD), and Kurtosis Ratio (KR2) and its variation (ΔKR2). ESG data were recorded from nine healthy participants at brachial and lumbar plexus sites with various electrode configurations. ATS consistently outperformed all other methods in suppressing cardiac artifacts of varying shapes. Although it did not fully preserve low-frequency content, ATS achieved the best balance between artifact removal and signal integrity. Algorithm performance improved when ECG contamination was lower, especially in brachial plexus recordings with closer reference electrodes.

## 1. Introduction

The functions of the spinal cord extend beyond the mere transmission of neural signals from the brain. It exhibits intrinsic information-processing capabilities and constitutes a substantial source of efferent signals with the potential to transform neural–machine interfaces (NMIs) [1]. The exploration of spinal cord activity can be approached through direct or indirect methods. Direct methods involve recording neural activity, while indirect methods analyze variations in vascular flow. Traditional recording techniques, though effective, are invasive and expensive. Fortunately, non-invasive and cost-effective alternatives are now within reach, such as functional near-infrared spectroscopy (fNIRS) [2] and high-density surface electromyography (HD-sEMG) [3].

It is important to acknowledge that recording spinal cord neuronal activity, known as electrospinography (ESG), via HD-sEMG is susceptible to artifacts induced by electrocardiographic (ECG) activity, similar to traditional electromyography (EMG) recordings in the trunk. The different ECG waves represent distinct phases of polarization and depolarization during each cardiac cycle, with the R peak being the main feature and the center of the ECG complex. Due to their high amplitude changes, these waves can be present in recordings of other biosignals. Removing the ECG component from EMG signals before analysis is crucial to preserve the integrity of the extracted characteristics, given the overlap in frequency components between the two signals.

To address this challenge, various algorithms have been developed, including high-pass filters, cardiac pattern subtraction, QRS gating, Wavelet transform, adaptive filters and blind source separation. Each technique offers distinct advantages and limitations. High-pass filters, for instance, can effectively remove low-frequency ECG components but may also eliminate relevant EMG information [4]. Cardiac pattern subtraction and QRS gating target specific cardiac artifacts, but require precise identification of cardiac events [5]. Wavelet transform and adaptive filters offer flexibility, but can be computationally intensive [6,7]. Blind source separation techniques attempt to isolate and remove ECG signals without prior information, but their effectiveness depends on the complexity of the signal environment [8].

A precise frequency window of interest of the ESG signals has not been well-established. Some studies have utilized signals ranging from 0.5 Hz to 30 kHz [9,10], whereas ECG signals reach frequencies up to 100 Hz and exhibit higher amplitude values than EMG [11]. Consequently, akin to the EMG, there is a necessity to eliminate ECG artifacts from ESG signals before conducting a thorough analysis. However, due to the novelty of ESG signal acquisition using HD-sEMG, limited work has been done to evaluate denoising techniques specifically for these signals.

Given the similarities between ESG and EMG in terms of artifact sources, this study evaluates the effectiveness of seven established ECG artifact removal algorithms, originally developed and validated for EMG signal processing, when applied to ESG data. The analysis focuses on the differences between the two signal types, particularly in their frequency components of interest and amplitude characteristics, which may influence algorithm performance. The objective is to evaluate the denoising effectiveness of these algorithms and determine their suitability for ESG signal analysis.

## 2. Materials and Methods

### 2.1. Participants

Nine able-bodied subjects (31.8 ± 9.1 years old) participated in the study, comprising four females and five males. None of the participants reported any known diseases or movement impairments and all were right-foot dominant. The participants were provided with information about the experimental procedure and signed a written informed consent form, as well as consent for video and image recording.

The study was carried out in accordance with the Declaration of Helsinki of the World Medical Association and was approved by the Office of Responsible Research at the Miguel Hernández University of Elche (DIS.JAP.09.21).

Experimental sessions were conducted over multiple days and not all participants took part in every experimental condition. Of the nine total participants, seven completed a single recording of experimental condition 1, while five completed a single recording of experimental condition 2 and one participant completed this condition twice. A detailed summary of the participation involved in each experiment analyzed in this study is presented in Table 1.

### 2.2. Devices

ESG signals were recorded using HD-sEMG equipment with a 2 kHz sampling frequency. Signals were amplified using Sessantaquattro+ equipment (OT Bioelettronica, Turin, Italy) and acquired through OTBioLab+ software (OT Bioelettronica, Italy). The device was positioned on the participant’s back and secured with an adjustable strap, while a reference electrode was placed on the right scapula. To ensure optimal signal conductivity, the electrode application area was cleaned using a dermabrasive gel followed by ethyl alcohol.

**Figure 1:**
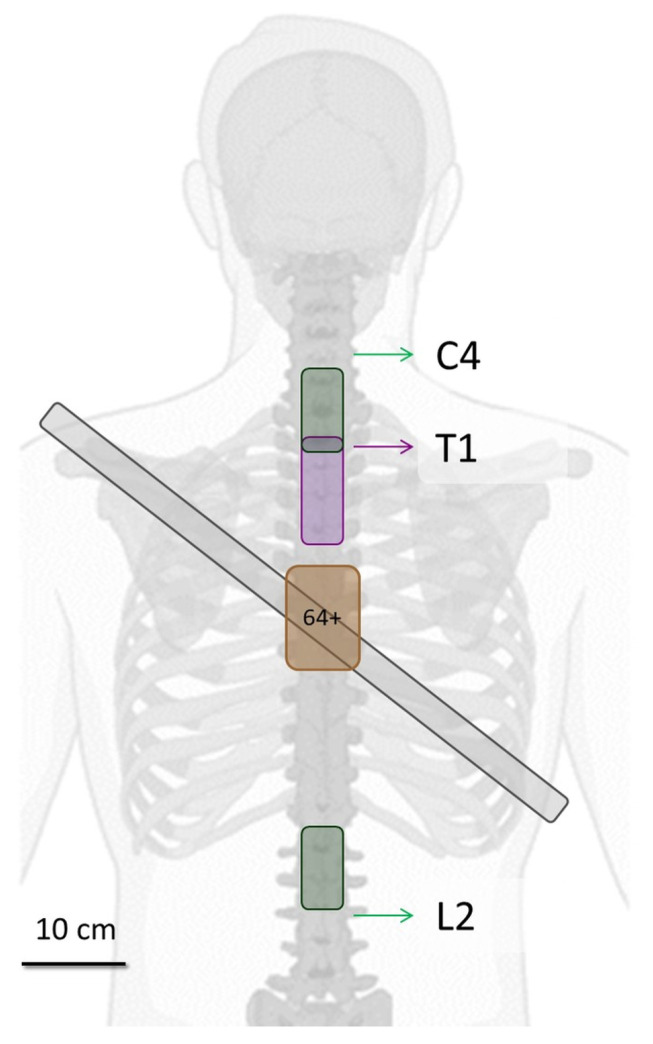
Placement of HD-sEMG matrix electrodes to record ESG and the vertebrae used as fiducial points for positioning, along with the placement of the Sessantaquatro+ device on the participant’s back. The matrix highlighted in purple corresponds to the first experimental configuration, while the green ones represent the second configuration.

Two distinct electrode configurations were employed, each designed to meet specific research objectives focused on recording activity from the brachial or lumbar plexus, regions where the nerves responsible for controlling the upper and lower limbs bifurcate. The placement of the electrodes was guided by anatomical fiducial points identified through palpation of the spinous processes at the C4, T1 and L2 vertebrae, as illustrated in Figure 1.

The experimental configurations were defined as follows:

- First Experiment: A matrix comprising 64 gold-coated electrodes (each with a 1 mm diameter) was arranged in a 13 × 5 configuration with an inter-electrode distance (IED) of 0.8 cm. This matrix was positioned over the T1 vertebra, with the first electrode aligned over the intervertebral disc directly beneath the T1 vertebra. This configuration was designed to capture activity originating from the brachial plexus (BP).
- Second Experiment: Two separate electrode matrices, each containing 32 gold-coated electrodes (1 mm diameter), were arranged in an 8 × 4 configuration with a 1 cm IED. The first matrix was positioned over the BP using the C4 vertebra as a reference point, with its second electrode positioned over the intervertebral disc immediately below the C4 vertebra. The second matrix was placed over the lumbar plexus (LP), aligned with the L2 vertebra, with its second electrode aligned over the intervertebral disc situated above the L2 vertebra.

### 2.3. Procedure

The experimental procedure comprised two recording trials. During each trial, participants stood with their eyes open for two minutes to record resting spinal cord activity, enabling the assessment of denoising performance under basal conditions. At the beginning of each recording, an initial 15-s waiting period was included to allow for the application of filters necessary for subsequent signal analysis.

The dataset employed in this study has been included in Zenodo [12]. The data used corresponds to the Stage 1 trials from both Experiment 1 and Experiment 2.

All analytical procedures were conducted offline using the MATLAB interface, version 2023a, and are publicly available on GitHub [13].

## 3. Denoising Algorithms

To the best of our knowledge, no filtering algorithms have yet been specifically developed or validated for non-invasively recorded ESG signals. However, because the principal artifact, ECG interference, is also found in EMG recordings, this study explores the applicability of established EMG denoising techniques to ESG data, taking into account potential differences in performance arising from the distinct characteristics of EMG and ESG signals. Specifically, seven ECG artifact removal algorithms originally developed and validated for EMG are evaluated for their denoising performance in the context of ESG signals.

Prior the implementation of the various algorithms, the locations of the R peaks within the ESG signals were determined using the Pan and Tompkins algorithm [14], a well-established technique for detecting QRS complexes in ECG signals. The selected denoising algorithms were sourced from [15].

A concise overview of each algorithm assessed in this study is provided below. For more detailed technical information, readers are referred to the comprehensive descriptions available in [16].

### 3.1. Template Subtraction (TS)

The Basic Template Subtraction method aims to eliminate ECG interference from EMG measurements through a series of steps. First, after the identification of the ECG beat peak locations, the positions are updated by calculating an average beat across the dataset, realigning peak positions. This step is essential due to variations in QRS complex detection. The ECG subtraction template for each beat is created by averaging 40 ECG beats around the current beat, centered at the R peak with a ±200 ms window that encompasses the prominent Q, R and S waves. Given that the participants exhibited an average heart rate of 82 ± 13 beats per minute, this corresponds to a minimum recording duration of approximately 30 s. The template is aligned with the current beat using cross-correlation and its amplitude and offset are adjusted through linear regression. This process is iteratively applied to each detected ECG beat to remove cardiac artifacts, resulting in a cleaned signal.

### 3.2. Adaptive Template Subtraction (ATS)

The classical template subtraction method often struggles to completely remove ECG interference from EMG signals due to the substantial amplitude difference between the ECG and EMG signals and the changing characteristics of ECG peaks over time [17]. To address these issues, an enhanced version of the template subtraction algorithm, called adaptive template subtraction (ATS) was proposed. ATS makes two key modifications to the original method: each ECG beat is divided into three segments (P, QRS and T) with their amplitudes adjusted separately to better match the current beat and the QRS segment is scaled in time to align with the QRS complex of the current beat, which significantly impacts signal distortions. In ATS, a subtraction template is constructed from 40 beats centered around the current beat. This template is then divided into three segments and 21 different time-scaled versions of the middle, QRS, segment are generated by stretching or shrinking. These templates are correlated with the current ECG beat and each segment is offset- and gain-adjusted using linear regression. The subtraction template with the lowest sum of squared differences from the current beat is chosen and subtracted from the signal to yield the cleaned EMG measurement.

### 3.3. High-Pass Filter with 200 Hz Cutoff Frequency (HP200) and Adaptive Template Subtraction and Subsequent High-Pass Filtering (ATS + HP200)

High-pass filtering is a common method for ECG interference removal, but its effectiveness depends on the cutoff frequency. A 30 Hz cutoff did not yield satisfactory results due to low signal-to-noise ratio (SNR) near the heart [4]. In the study of Petersen et al. [16], a fourth-order Butterworth high-pass filter with a 200 Hz cutoff (HP200) and a combination of the ATS algorithm with HP200 (ATS + HP200) performed reasonably well. Thus, the two options were included in the list of algorithms analyzed in this research.

### 3.4. Stationary Wavelet Transform (SWT)

The application of the wavelet denoising technique, introduced by Donoho [18], is a versatile method for separating noise from signals. Several parameters, such as decomposition depth, wavelet family and thresholding, influence the denoising process. For ECG-EMG mixtures, the 4-tap Daubechies wavelet (db2) with at least three levels of decomposition and a level-dependent fixed threshold of 4.5 σk (EMG variance estimate at level k) is recommended. The option used in this study and presented in [16] proposed three key modifications. Firstly, it advocates the use of the stationary wavelet transform (SWT) for noise reduction due to its superior capabilities compared to classical wavelet transforms. Secondly, it suggests the application of hard thresholding, as opposed to soft thresholding, for ECG removal to achieve a more accurate reproduction of R peak amplitudes. Lastly, it recommends substituting the default median value used for noise variance estimation with a moving median filter. This modification takes into account fluctuations in the EMG noise level during recording and specifically excludes R peaks from the median filter. This approach minimizes the influence of ECG coefficients on noise estimation.

### 3.5. Second Order Extended Kalman Smoother (EKS2)

McSharry et al. presented a model-based filtering approach for ECG signal separation [19]. The model represents the ECG waveform through a mixture of Gaussian curves and introduces quasiperiodicity into the signal by orbiting around the unit cycle over time. Each ECG peak is associated with a specific angle of the unit cycle. The system estimation is executed through an extended Kalman filter (EKF) and extended Kalman smoother (EKS). The states of the system include the rotation angle and the generated ECG signal, while other parameters (e.g., peak locations, heart rate, Gaussian wave parameters) are treated as Gaussian independent and identically distributed (i.i.d.) process noise. Variations of this method have been proposed and the one employed in this paper is the filtering method termed the EKF2/EKS2, with the number “2” denoting the count of estimated states. This variation includes several techniques to enhance numerical stability, such as sequential filtering, Joseph stabilization, state constraints and Rauch–Tung–Striebel smoothing [20]. These modifications serve to reduce the occurrence of outliers and to enhance the overall filtering process.

### 3.6. Empirical Mode Decomposition (EMD)

The EMD algorithm constitutes a data-driven method for signal decomposition in the time domain [21]. Various EMD-based denoising strategies have been proposed for single-channel physiological measurements. These strategies encompass adaptive filtering, independent component analysis, canonical correlation analysis and techniques that resemble wavelet denoising. In cases characterized by strong ECG interference, empirical observations [22] have indicated that the EMG signal predominantly resides in the first Intrinsic Mode Function (IMF), while the ECG component is primarily present in higher IMFs.

## 4. Metrics for Quantitative Evaluation

In the comparative assessment of algorithmic performance, six distinct quantitative metrics were employed, based on existing comparative studies on the denoising of EMG signals [11,23]. It is important to highlight that unlike numerous prior studies evaluating algorithms for ECG component removal, where uncontaminated signals were available for assessment, this study exclusively analyzed signals recorded in a contaminated state. To the best of our knowledge, ESG signals have not been recorded non-invasively without ECG contamination. Consequently, the exact characteristics of these signals are not well known, which complicates the generation of simulated signals that could be used to validate the algorithms as a “ground truth.” Nevertheless, it is worth noting that these algorithms have previously been validated using simulated EMG signals. For the purposes of this study, the signals obtained after the initial preprocessing stage, common to all algorithms, were designated as the reference signals for subsequent comparisons. This preprocessing consisted of applying a second-order Butterworth band-pass filter (10–500 Hz) to remove DC offsets and high-frequency noise. Additionally, power-line interference at 50 Hz and its harmonics (up to 500 Hz) was attenuated using third-order Butterworth notch filters. A limitation of this approach is that the metrics may be influenced by using these filtered signals as the reference. To mitigate this effect, the performance metrics should be interpreted holistically, as individual measures could lead to misleading conclusions. For instance, an algorithm that leaves the recorded signal unaltered would yield optimal *RE*, *CC* and *SD* values, yet the signal would still contain ECG artifacts, undermining the validity of the results. Conversely, an algorithm that completely nullifies the signal would achieve an ideal *SNR*, while a random normal sequence could theoretically produce an optimal *KR2* value. Therefore, in this study, all metrics should be considered simultaneously to provide a balanced and reliable assessment of algorithm performance.

The computed variables were obtained from a 120-s segment of the signal, excluding the initial 15 s. These measures were repeated for each trial, electrode and subject and subsequently averaged, with differentiation made between the BP and LP matrices. Electrodes that experienced connection issues and recorded a value of zero throughout the entire session were considered null.

The metrics utilized for evaluating the performance of the various algorithms are detailed below:

### 4.1. Relative Error (RE)

Relative Error (*RE*) quantifies the discrepancy between the power spectral densities (*PSD*) of the original signal, $y_{0}$, and the processed signal, $y_{1}$, obtained after applying different algorithms. A lower *RE* value indicates superior algorithmic performance.

(1) $$RE=\frac{\sum {\left(PSD\left(y_{0}\right)-PSD\left(y_{1}\right)\right)}^{2}}{\sum {\left(PSD(y_{0})\right)}^{2}}$$

### 4.2. Signal-to-Noise Ratio (SNR)

The Signal-to-Noise Ratio (*SNR*) serves as a crucial assessment criterion, considering that the mean of EMG or ESG signals is typically zero. *SNR* is computed using the standard deviations of $y_{0}$ and $y_{1}$. Higher *SNR* values indicate improved algorithmic performance.

(2) $$SNR=10\times \log_{10}\left(\frac{var(y_{0})}{var(y_{1}-y_{0})}\right)$$

### 4.3. Cross-Correlation (CC)

Cross-Correlation (*CC*) assesses the degree to which the estimated signal retains the shape and amplitude of the original signal. Higher *CC* values indicate greater fidelity in signal preservation.

(3) $$CC=100\times \frac{\sum y_{0}\times y_{1}}{\sqrt{\sum y_{0}^{2}\times \sum y_{1}^{2}}}$$

### 4.4. Spectral Distortion (SD)

Spectral Distortion (*SD*) measures the extent to which the estimated signal preserves the frequency content of the original signal. *SD* was computed for two distinct frequency ranges: 0–20 Hz and 20–200 Hz, enabling a detailed analysis of the impact of the ECG component on the lower frequency range.

(4) $$SD=100\times \frac{PSD(y_{1})}{PSD(y_{0})}$$

### 4.5. Kurtosis Ratio (KR2)

The Kurtosis Ratio (*KR2*) is a robust measure of kurtosis that has been proposed as an indicator of ECG interference in EMG signals [24]. This metric evaluates the distribution of the estimated signal in comparison to a standard Gaussian distribution. The formulation of the *KR2* employed in this study was introduced in [25] and it is computed using the inverse cumulative distribution function, denoted in the equation as $F^{-1}$, for each analyzed signal. Higher absolute values of *KR2* indicate a greater degree of ECG interference [24].

(5) $$KR2=\frac{F^{-}1\left(0.975\right)-F^{-}1\left(0.025\right)}{F^{-}1\left(0.75\right)-F^{-}1\left(0.25\right)}-2.91$$

### 4.6. Variation of Kurtosis Ratio (ΔKR2)

Since the relevance of *KR2* in the filtered signals depends on the initial *KR2* value in the raw signal, this metric can also be expressed as the variation of *KR2*. This approach provides a more robust measure across comparing different subjects.

(6) $$\Delta KR2=\frac{|KR2_{y_{1}}|-|KR2_{y_{0}}|}{|KR2_{y_{0}}|}$$

## 5. Results

This section presents the outcomes of the ECG artifact removal applied to ESG signals using seven different denoising algorithms. The evaluation follows two main approaches: first, a qualitative visual inspection of signal behavior in both the time and frequency domains, and second, a quantitative assessment based on established signal quality metrics. Results are reported across three electrode matrix configurations (64-electrode BP, 32-electrode BP and 32-electrode LP), allowing for a comparative analysis of algorithm performance under varying spatial and anatomical conditions. The findings aim to elucidate the effectiveness, limitations and signal preservation capabilities of each method in the context of ESG signal processing.

### 5.1. Time- and Frequency-Domain Signal Comparisons

**Figure 2:**
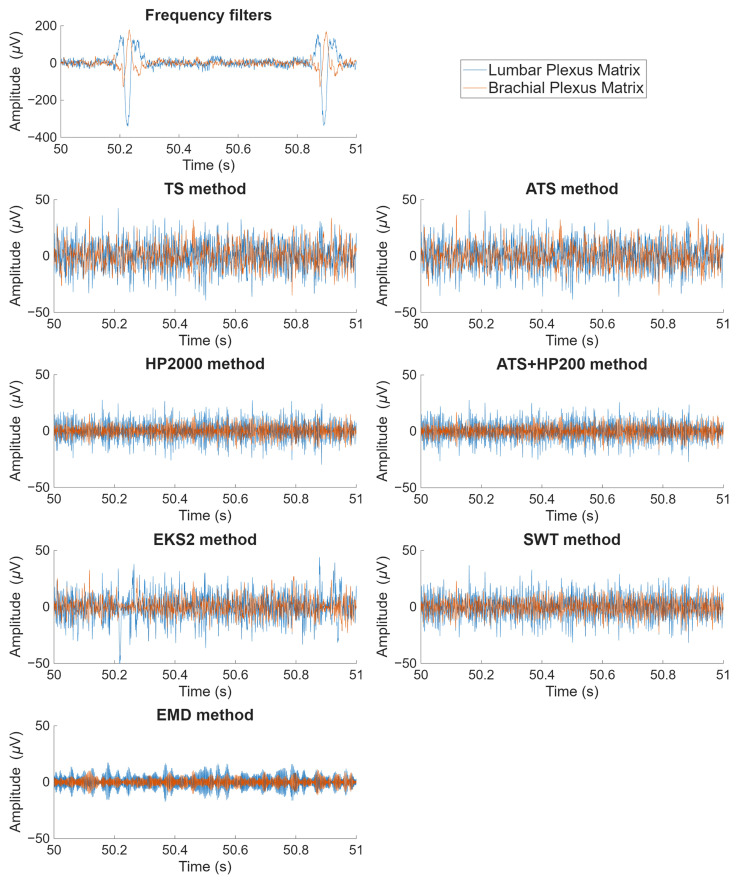
Time-domain representation of ESG signals, including the original signal after frequency filtering and the results obtained following the application of the seven examined algorithms: Template Subtraction (TS), Adaptive Template Subtraction (ATS), High-pass filtering with a 200 Hz cutoff frequency (HP200), Adaptive Template Subtraction followed by high-pass filtering (ATS + HP200), Second-order Extended Kalman Smoother (EKS2), Stationary Wavelet Transform (SWT) and Empirical Mode Decomposition (EMD). The signals correspond to the first electrode of both the lumbar and brachial plexus (LP and BP, respectively) matrices for the first trial of subject S03 in the second experiment. Notably, the amplitude range of the original signal is significantly wider. However, this representation was intentionally adjusted to ensure the comprehensive visualization of the complete morphology of the ECG artifact.

**Figure 3:**
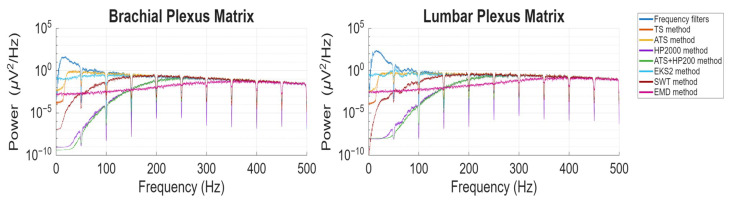
Frequency-domain representation of ESG signals, including the original signal after frequency filtering and the results obtained following the application of the seven examined algorithms: Template Subtraction (TS), Adaptive Template Subtraction (ATS), High-pass filtering with a 200 Hz cutoff frequency (HP200), Adaptive Template Subtraction followed by high-pass filtering (ATS + HP200), Second-order Extended Kalman Smoother (EKS2), Stationary Wavelet Transform (SWT) and Empirical Mode Decomposition (EMD). The signals correspond to the first electrode of both the brachial and lumbar plexus (BP and LP, respectively) matrices for the first trial of subject S03 in the second experiment.

Figure 2 and Figure 3 provide a visual representation of the final signals obtained after applying the seven algorithms under study, depicted in both the time and frequency domains, respectively.

In Figure 2, particular attention has been directed towards illustrating two QRS complexes, facilitating a comparative evaluation of the efficacy of ECG artifact elimination. While most algorithmic outputs appear similar, the extent of residual noise varies. Among the evaluated methods, EKS2 exhibits the most significant residual artifacts, likely due to the incomplete suppression of ECG wave components. This issue is particularly pronounced in the LP matrix, where the higher amplitude of artifacts poses additional challenges for the denoising process. The EMD algorithm constitutes a notable exception, as it produces an output that deviates substantially from the others, resembling an entirely distinct signal with unique tonal characteristics.

Regarding the frequency spectrum, illustrated in Figure 3, all algorithms exhibit a general tendency to converge towards the frequency-filtered signal, albeit at different frequency ranges. Both matrices demonstrate similar behavior, with the LP matrix containing a greater number of components below 50 Hz due to the higher amplitude of cardiac artifacts. Across both cases, the TS and ATS algorithms reach convergence first, achieving it within the 100–150 Hz range. EKS2 achieves similar values, but converging later in the BP matrix. The SWT method follows, reaching convergence at approximately 150–200 Hz. HP200 and HP200-ATS converge after their frequency cut-off, around 250 Hz, while the EMD algorithm reaches convergence at approximately 350–400 Hz.

A consistent trend across algorithms is the near-complete elimination of lower-frequency components before gradually restoring the characteristics of the original signal. This behavior can be attributed to the removal of frequency components associated with cardiac artifacts. However, EKS2 diverges from this pattern, as its lower-frequency components are never fully suppressed and, in some instances, even exceed the amplitude of the original signal, an effect more pronounced in the LP matrix. These lower-frequency components likely correspond to the residual cardiac artifacts observed in the time domain. The EMD method exhibits a unique spectral morphology, lacking clear suppression of lower-frequency components. This distinct behavior is evident in both time and frequency-domain representations.

### 5.2. Quantitative Evaluation

Table 2 presents the mean values and standard deviations of the computed variables across the electrodes of each matrix configuration, encompassing two distinct trials per subject and their corresponding averages. While the overall performance of the algorithms is generally consistent with previous observations based on EMG signals, certain cases exhibit suboptimal adaptation, resulting in signal distortion or artifact amplification rather than effective denoising. These differences in algorithm performance may be attributed to the inherent distinctions between EMG and ESG signals.

**Figure 4:**
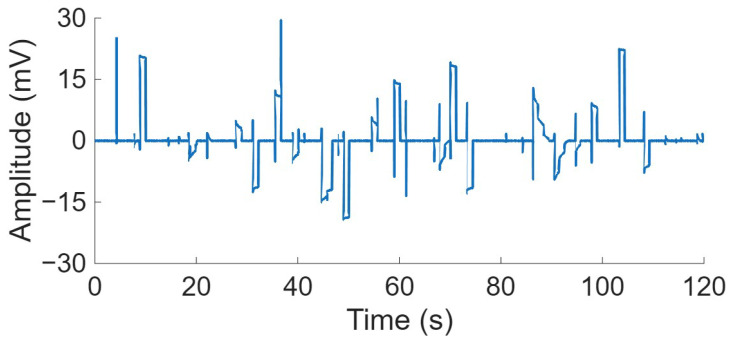
Time representation of an ESG signals filtered with the EKS2 algorithm corresponding to the electrode 11 from the brachial plexus (BP) matrix, recorded during the first trial of subject S07 in the second experiment.

After observing high variability in certain cases, a more detailed analysis was conducted on individual trials. Instances were identified in which the filter appeared not to have achieved convergence, producing amplitude values in the millivolt range, substantially exceeding the typical amplitude range of the other electrodes, measured in µV. Figure 4 illustrates an example in which the signal amplitude in some instances reaches the order of tens of millivolts, while in others it remains comparable to the original raw signal, within the microvolt range.

To systematically assess these occurrences and their impact on the remaining data, cases exhibiting a failure to achieve convergence were examined, while excluding instances of inefficient denoising by the algorithms. Such events were designated as “convergence errors”. To address these anomalies, a convergence error elimination process was implemented using a thresholding approach applied to *KR2* values across all electrodes, subjects and matrix configurations. The *KR2* values obtained prior to any ECG denoising were used as reference limits to define the extreme bounds of expected signal behavior. The upper threshold was defined as the mean *KR2* value computed across all combinations of the frequentially filtered signal, while the lower threshold was set at the 15th percentile, with its sign inverted. These thresholds were determined through an exploratory scanning procedure, with the objective of excluding only extreme deviations rather than discarding results reflecting generally suboptimal performance. This thresholding strategy aimed to identify and remove cases in which the filtering process failed to effectively suppress cardiac artifacts, instead either amplifying them or altering the signal in a way that caused *KR2* values to deviate substantially from expected norms.

Table 3 summarizes the number of convergence errors detected for each algorithm across the different matrix configurations. Convergence errors were observed in the EKS2 and EMD algorithms, with EKS2 affecting all matrices and EMD predominantly impacting the 32-electrode matrixes. In the case of EKS2, discarding these instances led to a notable reduction in standard deviations, whereas for EMD, the effect on the average outcome was minimal. Given the low frequency of these occurrences, around 0.22% and 0.67% of the total data for the affected algorithms, it was decided to report in Table 2 the values obtained after excluding the trials affected by convergence errors. This approach allows for a fair comparison of the algorithms, as the overall performance remains representative, while a few extreme cases would otherwise disproportionately influence the mean performance. Nevertheless, it should be noted in the final conclusions that, if either the EMD or EKS2 algorithm were to be selected for practical use, an additional processing step would be required to mitigate the occurrence of convergence errors.

**Figure 5:**
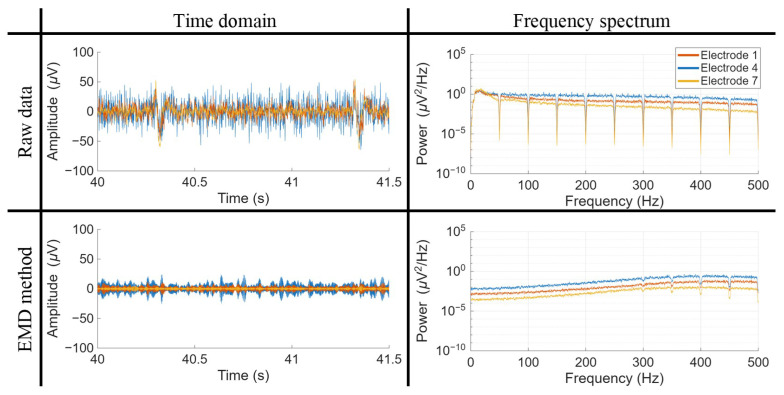
Time and frequency domain representation of ESG signals, comparing the frequency-filtered signal with the signal denoised using the EMD method. The figure highlights three electrodes from the same column of the brachial plexus (BP) matrix during the second trial of subject S02 in the second experiment.

For the EMD method, despite significant deviations in the Δ*KR2* metric, only four instances out of the 896 data points across the 32-electrode matrices exhibited convergence errors, representing less than 0.5% of the cases. To investigate the sources of high variability in these values, Figure 5 showcases an example featuring three electrodes from the same row of the BP matrix. In the raw signals, these electrodes display similar amplitudes for components below 50 Hz while maintaining consistent frequency differences at higher ranges. After EMD filtering, these frequency differences persist across the entire spectrum, resulting in time-domain signals characterized by distinct tonal elements. *KR2* values for these electrodes remain within the predefined limits derived from the frequentially filtered signals and are therefore not classified as convergence errors. However, when considering the relative variation of *KR2* values compared to their corresponding pre ECG-denoising signals, some electrodes exhibit substantial changes, such as electrode 4 in this example, which shows an increase in *KR2* of 1359.81%. This observation suggests that the variability in Δ*KR2* does not indicate extreme behavior following EMD processing, but rather reflects the inherent tendency of this filtering method to distort the signal, thereby deviating from the characteristics of the original waveform.

**Figure 6:**
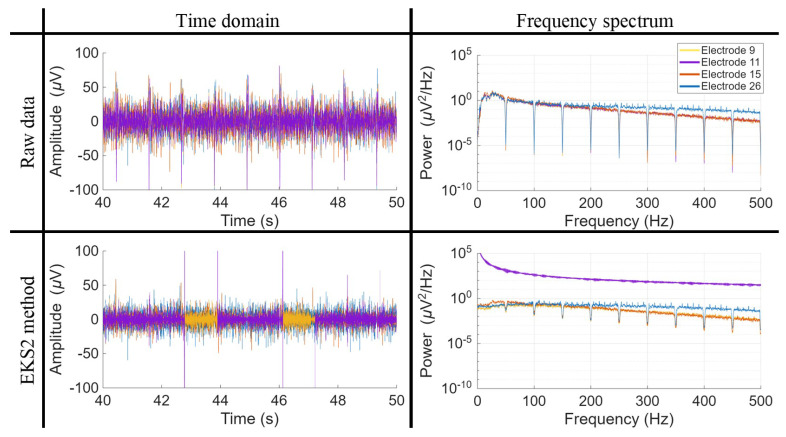
Time and frequency domain representation of ESG signals, comparing the frequency-filtered signal with the signal denoised using the EKS2 method. The figure highlights three electrodes from the same column, along with an additional electrode from the brachial plexus (BP) matrix, recorded during the first trial of subject S07 in the second experiment. Notably, electrode 11 exhibits significant amplitude deviations, reaching values in the range of mV, caused by a lack of convergence in certain time intervals.

The EKS2 method identified 12 convergence errors across all matrix configurations, totaling 1792 data points, which represents slightly more than 0.5% of the cases. In this instance, the metrics exhibiting higher variability, both *SD* measures, are effectively corrected through the elimination of convergence errors. To illustrate this behavior, Figure 6 displays some electrodes from the 32-electrode BP matrix. In the raw signal, differences between electrodes are minimal, mostly due to electrode 26, which comes from a different column and only shows differences at frequencies above 200 Hz. After filtering, EKS2 behaves inconsistently: some electrodes, like electrode 15, still have residual artifacts, while others, such as electrode 11, show large divergences with signal amplitudes reaching millivolt levels. Electrode 11 is thus classified as an error based on *KR2* values. Unlike the EMD case, in the EKS2 method the observed high variability arises from genuinely extreme signal behavior following the ECG denoising process, although the overall performance of the algorithm remains well adapted to the ESG signal characteristics.

### 5.3. Metric-Based Performance by Algorithm

Before comparing the computed metrics for each algorithm, presented in Table 2, normality and homogeneity of variance were assessed using the Kolmogorov–Smirnov and Levene’s tests, respectively. It was determined that, in most cases, the assumption of normality could not be excluded (*p* < 0.05) and the hypothesis of equal variances across different groups was rejected (*p* < 0.01). As a result, non-parametric statistical methods were employed for further analysis. A Friedman test was conducted to assess the performance of the seven different algorithms, revealing statistically significant differences across all seven metrics and three matrix configurations (*p* < 0.01). Following a Wilcoxon rank sum test evaluating the differences among each pair of algorithms for each metric. Although the metrics are initially compared individually, it is important to note that the performance of a single metric is not conclusive on its own, as it may be influenced by the specific limitations of each algorithm. Therefore, a holistic evaluation of all metrics is essential to accurately identify and interpret such cases.

*RE*: Across all matrices, the ATS method demonstrated the lowest *RE* values, consistently outperforming other algorithms. For the 64-electrode BP matrix, ATS achieved an *RE* of 0.12 ± 0.03, maintaining its superior performance after convergence errors removal. Similar trends were observed in the 32-electrode matrices, where ATS yielded an *RE* of 0.11 ± 0.02 for the BP and 0.15 ± 0.04 for the LP. Although TS exhibited similar mean performances across all matrix configurations, the difference among algorithms in the 32-electrode BP matrix was not statistically significant (*p* = 0.0764), whereas, in the remaining configurations, significance was confirmed (*p* < 0.01), with small effect size (|r| ≈ 0.3). In contrast, HP200 and ATS + HP200 resulted in substantially higher *RE* values, exceeding 1.0. While both algorithms performed similarly, ATS + HP200 was statistically the worst across all matrix configurations (*p* < 0.01, |r| > 0.8).

*SNR*: The highest *SNR* values were observed with TS across all BP configurations. TS achieved an *SNR* of 0.98 ± 0.73 and 1.34 ± 1.08 in the BP matrix of 64 and 32 electrodes, respectively. While slightly outperforming ATS, their mean values were comparable, with no statistically significant difference observed (*p* > 0.01). In the LP matrix, EKS2 yielded the highest *SNR* (0.23 ± 0.59), followed by TS (0.20 ± 0.36), which was significantly lower (*p* = 0.0126). On the other hand, EMD was the algorithm that produced significantly lower *SNR* values (*p* < 0.01, |r| > 0.3).

*CC*: TS also achieved the highest *CC* percentages, indicating superior preservation of the original signal morphology. In the 64-electrode BP matrix, TS attained a *CC* of 54.68 ± 17.33% following convergence errors elimination. Similarly, in the 32-electrode BP matrix, TS maintained a *CC* of 60.10 ± 20.24%, although EKS2 emerged as the best-performing algorithm for the LP matrix with 24.45 ± 14.73%. TS followed closely with 22.94 ± 15.68%, which was statistically lower (*p* = 0.0034), but with a small effect size (|r| = −0.1016). Once more, TS and ATS exhibited similar mean performances with no statistically significant difference (*p* > 0.01). Conversely, EMD produced the lowest *CC* values (*p* < 0.01, |r| > 0.3), once again indicating significant signal alteration.

*SD*: *SD* was analyzed separately for low (0–20 Hz) and high (20–200 Hz) frequency bands. Within the lower frequency range, EKS2 exhibited the highest *SD* across all matrix configurations, despite its limited denoising effectiveness. This seemingly favorable performance is primarily due to the algorithm’s preservation of lower frequencies, which also results in the retention of certain artifact components. In the 64-electrode BP matrix, EKS2 achieved an *SD* of 17.45 ± 19.43%, while for the 32-electrode matrices, values of 24.08 ± 31.37% and 12.81 ± 69.52% were recorded for the BP and LP, respectively. In the higher frequency range, TS demonstrated the highest *SD* values across all matrices. The 64-electrode BP matrix yielded an *SD* of 78.99 ± 14.31%, while the 32-electrode matrices exhibited *SD* values of 78.58 ± 15.41% and 42.93 ± 21.43% for the BP and LP, respectively. In both frequency bands, HP200 and ATS + HP200 were the least effective, as the applied filter nearly eliminated frequency components below the cut-off threshold, particularly for frequencies below 20 Hz. However, performance differences between these two methods were not statistically significant (*p* > 0.01).

*KR2*: The lowest absolute *KR2* values were obtained using ATS + HP200 for the 64-electrode matrix (0.06 ± 0.07), whereas EMD produced the lowest values for the 32-electrode matrices (−0.05 ± 0.24 and −0.01 ± 0.23 for the BP and LP, respectively). However, these results are strongly influenced by the high variability and large standard deviation, with some values differing even in sign. This behavior can be attributed to the tendency of the EMD algorithm to distort the original signal, as it does not appear to fully adapt to the inherent characteristics of the ESG signals. Notably, when considering the mean absolute *KR2* value, both matrices yield a value of 0.18. For the 64-electrode BP matrix, the algorithms TS, ATS and SWT, as well as HP200 and ATS + HP200, exhibited comparable performance within their respective groups although statistically significant differences were observed between the two groups (*p* < 0.01, |r| < 0.1). In contrast, for the 32-electrode BP matrix, all five algorithms demonstrated similar performance, with no statistically significant differences among them (*p* > 0.01). In the LP matrix, however, the grouping of algorithms with similar performance differed, with TS and ATS forming one group and HP200, ATS + HP200 and SWT forming another. Within each group, no statistically significant differences were observed among the respective algorithms (*p* > 0.01), while they were found between algorithms from different groups (*p* < 0.01, |r| < 0.3).

Δ*KR2*: Regarding Δ*KR2*, the most significant reductions were observed for ATS + HP200 in the 64-electrode BP and 32-electrode LP matrices, with values of −0.94 ± 0.08 and −0.96 ± 0.07, respectively. Similarly, SWT demonstrated substantial reductions in the 32-electrode BP matrix, reaching −0.86 ± 0.32. However, overall performance among the algorithms was relatively comparable. In both BP matrices, TS, ATS, HP200, ATS + HP200 and SWT exhibited no statistically significant differences (*p* > 0.01), except for the comparison of SWT with HP200 (*p* = 0.0011) and ATS + HP200 (*p* = 0.0015) in the 64-electrode matrix with a minimal effect size (|r| < 0.1). In the LP matrix, the groups with statistically similar performances included TS, ATS and EMD, as well as HP200, ATS + HP200 and SWT.

A notable exception is the EKS2 algorithm, where the strong preservation of lower frequencies reflects ineffective denoising. This effect is particularly pronounced in the 32-electrode BP matrix, where positive Δ*KR2* values (18.42 ± 349.72 before convergence error elimination and 1.18 ± 12.82 after) further highlight the need for additional processing to mitigate these distortions. Although convergence error cases account for less than 1% of the total cases, the degree of signal distortion they introduce is substantial enough to warrant further investigation and may render this algorithm initially incompatible with ESG data. Additionally, EMD exhibits poor performance in terms of Δ*KR2*, showing the highest deviation among all algorithms. This suggests that, although the processed signals may present *KR2* values comparable to those of other algorithms, their structural integrity is significantly altered relative to the original signal, leading to markedly higher Δ*KR2* values.

### 5.4. Comparison Across Matrix Configurations

A comparative analysis was also conducted to evaluate the impact of different electrode configurations on signal processing outcomes. Differences in electrode placement relative to the heart and reference electrode can influence the susceptibility to ECG contamination, affecting the characteristics of ECG artifacts and, consequently, algorithm performance.

A Friedman test was performed to assess the performance of the three matrix configurations across seven metrics, revealing statistically significant differences in all cases (*p* < 0.05) except for *RE* in the HP200, SWT and EMD algorithms. Subsequently, a Wilcoxon rank sum test was conducted to examine pairwise differences between matrices for each metric and algorithm. The LP matrix was found to be significantly different from both BP matrices (*p* < 0.05) except for *RE* in the 64-electrode BP matrix. Effect size analysis showed |r| > 0.05 for *SNR*, *CC* and *SD* across all algorithms and |r| > 0.03 for Δ*KR2* across all algorithms only in comparisons between the BP and LP matrices with 32 electrodes.

Comparisons between the two BP matrices revealed smaller, yet statistically significant differences (*p* < 0.05) in most cases, with the exception of *RE* for the ATS and EMD algorithms, *SNR* and *CC* for SWT, *SD* for frequencies above 20 Hz for TS, ATS and EKS2 and Δ*KR2* for EMD, with |r| < 0.03. Although both matrices were positioned above the BP, they differed in reference placement (C4 vs. T1), resulting in approximately 10 cm of separation. Consequently, minor variations in recorded signals were expected, although their effect size was small.

In contrast, when compared to the LP matrix, these differences became more pronounced, with effect size ranging from moderate to large. This disparity is likely attributable to the increased ECG interference in the LP matrix, which affects both the amplitude and temporal characteristics of the signals. The greater effort required to suppress these interferences led to reduced preservation of the original signal and higher *KR2* values. Nonetheless, since the raw signals also exhibited elevated *KR2* values, the resulting Δ*KR2* values were comparatively more favorable in the LP matrix.

Overall, while subtle differences exist between the two BP configurations, the LP matrix consistently presents greater challenges due to higher ECG contamination. These results highlight the importance of electrode configuration and reference placement in ESG studies, particularly when selecting or evaluating denoising algorithms.

## 6. Discussion

The performance of artifact removal algorithms varied across different metrics, highlighting distinct strengths and trade-offs. ATS consistently achieved the lowest *RE* values, EKS2 demonstrated the best preservation of spectral characteristics in the low-frequency range, while TS excelled in preserving high-frequency components. Additionally, TS outperformed other algorithms in terms of *SNR* and *CC*, particularly for the BP matrices, taking EKS2 the lead for the LP matrix. For metrics related to *KR2*, the most effective approaches where ATS + HP200 and EMD for absolute *KR2* values, while ATS + HP200 and SWT resulted in the most substantial reductions in Δ*KR2*. These findings emphasize the importance of selecting an artifact removal algorithm based on the specific research objectives, as different methods offer distinct advantages.

A key limitation of this study is the absence of an uncontaminated reference signal for comparison. Consequently, the employed performance metrics should be interpreted holistically, as individual assessments may lead to misleading conclusions.

Despite EMD achieving the lowest mean absolute *KR2* value, this result is influenced by substantial variability, including values with opposite signs. Furthermore, the processed signals exhibited significant deviations from the original signal, as reflected in the lower *CC* and *SD* values, as well as high values of Δ*KR2*. Due to these inconsistencies, EMD is deemed unsuitable for ESG signal denoising.

Similarly, EKS2 is also considered unsuitable. Although this algorithm excels in frequency preservation, it is ineffective in artifact removal, resulting in the highest absolute *KR2* values for all three matrices. Additionally, in approximately 1% of cases, the algorithm exhibited instability during filtering, producing convergence error data.

The remaining algorithms demonstrated comparable performance in certain instances (*p* > 0.05), particularly for *KR2*-related metrics. These results are consistent with those reported in state-of-the-art cardiac artifact filtering methods for EMG signals, which typically range from 0.01 to 1.43 [11]. Notably, the values obtained in this study fall within the lower end of this spectrum. However, in terms of frequency preservation, TS and ATS demonstrated significantly superior performance. While TS achieved the best results more frequently, ATS exhibited better preservation of low-frequency components (*p* < 0.05), an important characteristic given the absence of a predefined frequency range of interest in ESG analysis. Since ATS performed similarly to TS in all other metrics (*p* > 0.05), except for *RE* in the 64-electrode BP and LP matrices, it can be concluded that ATS is at least as effective as TS across all evaluated metrics.

**Figure 7:**
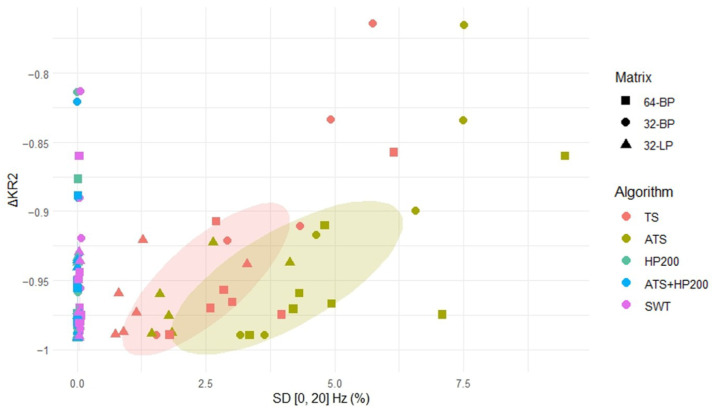
Representation of the Spectral Distortion (SD) for frequencies below 20 Hz versus the variation of Kurtosis Ratio (ΔKR2) across the following algorithms: Template Subtraction (TS), Adaptive Template Subtraction (ATS), High-Pass Filtering with a 200 Hz cutoff frequency (HP200), ATS combined with High-Pass Filtering (ATS + HP200) and Stationary Wavelet Transform (SWT). The comparison distinguishes between the three matrix configurations: the 64-electrode brachial plexus (BP) matrix, the 32-electrode BP matrix and the 32-electrode lumbar plexus (LP) matrix. Additionally, an ellipse encompassing 50% of the data for each algorithm has been included for further clarity.

In summary, the five algorithms yielded similar mean *KR2* and Δ*KR2* values, as shown in Table 2, with TS and ATS performing slightly worse in these metrics. In contrast, performance differed more markedly with respect to spectral preservation. As shown in Figure 7, where *SD* values are plotted for frequencies between 0 and 20 Hz, HP200, ATS + HP200 and SWT demonstrated suboptimal performance, while TS and ATS, particularly the latter, achieved superior results. A notable inverse relationship was observed between spectral preservation and kurtosis-based metrics. As illustrated in Figure 7, more aggressive denoising approaches that suppress frequency components more effectively tend to produce lower *KR2* values, suggesting stronger artifact attenuation.

Furthermore, differences among matrix configurations were observed; while the BP matrices produced values within a similar range, the LP matrix exhibited lower *SD* and Δ*KR2* values. This finding aligns with previous observations that cardiac artifacts are more prominent in LP recordings, leading to a greater loss of original signal components during denoising.

It is important to note that these conclusions are derived from a specific ESG dataset recorded under static standing conditions, characterized by minimal movement and muscular activity. ESG signal properties, particularly reflecting the variability in ECG artifact characteristics, can vary substantially depending on recording parameters, electrode placement, subject anatomy and physiological variability. Nonetheless, the inclusion of both BP and LP electrode matrices, as well as data from nine subjects, allowed the results to be assessed under a range of recording conditions. Accordingly, while the present findings support ATS as the most robust algorithm under static conditions, its performance in dynamic contexts should be further verified. Preliminary tests conducted on additional movement trials, available in the dataset published on Zenodo [12], indicate that ATS maintained stable denoising performance without requiring additional tuning, suggesting promising generalizability to dynamic conditions.

Despite its advantages, ATS presents certain limitations, including relatively high computational cost and processing time. Although other algorithms analyzed in this study also face similar constraints, these considerations may influence their practical applicability. In cases where low-frequency preservation is less critical, the TS algorithm may serve as a suitable alternative due to its considerably lower computational demand. Furthermore, when high-frequency preservation is prioritized or more aggressive attenuation of ECG components is required, algorithms such as HP200, ATS + HP200, or SWT may be preferable, as they yielded lower *KR2* values and more extensive ECG suppression.

## 7. Conclusions

This study aimed to evaluate and compare seven algorithms for removing ECG contamination from ESG signals, a crucial step for accurate analysis of spinal cord neuronal activity. Given the absence of ESG-specific filtering methods for non-invasively recorded ESG signals, commonly used EMG denoising algorithms were tested to assess their applicability and effectiveness with ESG data. The assessment of these various algorithms was conducted through the utilization of seven performance indicators.

Upon a comprehensive analysis of the information gathered, the Adaptive Template Subtraction (ATS) algorithm emerged as the most effective among them, consistently delivering superior results regardless of the ECG artifact’s morphology introduced by different electrode matrix configurations. Notably, while this algorithm did not entirely preserve the low-frequency components of the original signal, it exhibited exceptional capabilities in thoroughly denoising the QRS complex. In a holistic view encompassing the entire frequency spectrum, it excelled in signal preservation. This attribute is expected to be particularly advantageous for future analyses, as it enables the examination of a wide spectrum of frequencies until a specific range of interest is identified.

Overall, while ATS demonstrated the most balanced performance across all metrics and conditions tested, the optimal choice of denoising algorithm should ultimately depend on the specific objectives of each ESG study and the spectral characteristics of the signals under investigation.

## Figures and Tables

**Table 1:** Distribution of the sessions carried out by each of the subjects for the three experiment configurations.

Subject	Experiment 1	Experiment 2
S01	14/11/2023	23/05/2024
S02	15/11/2023	11/06/2024
S03	04/12/2023	07/05/2024
S04	05/12/2023	
S05	18/12/2023	07/05/2024
S06	19/12/2023	
S07		27/06/2024 15/10/2024
S10		11/02/2025
S11	20/02/2025	

**Table 2:** Performance indicators, including Relative Error (RE), Signal-to-Noise Ratio (SNR), Cross-Correlation percentage (CC), Spectral Distortion percentage (SD), Kurtosis Ratio (KR2) and variation of KR2 (ΔKR2) were assessed for each of the evaluated algorithms. These algorithms encompassed Template Subtraction (TS), Adaptive Template Subtraction (ATS), High-pass filtering with a 200 Hz cutoff frequency (HP200), Adaptive Template Subtraction followed by high-pass filtering (ATS + HP200), Second-order Extended Kalman Smoother (EKS2), Stationary Wavelet Transform (SWT) and Empirical Mode Decomposition (EMD). For each performance metric, the results are presented as the mean ± standard deviation, calculated from data collected across all electrodes of the three matrices and seven subjects, with data derived from two separate trials. Specifically, section (64-BP) corresponds to the brachial plexus (BP) matrix with 64 electrodes, (32-BP) corresponds to the BP matrix with 32 electrodes and (32-LP) corresponds to the lumbar plexus (LP) matrix. Special attention was given to distinguishing results before and after convergence errors elimination, indicated by grey and white backgrounds, respectively. For algorithms where no convergence errors were identified, only the post-elimination values are reported. Additionally, to enhance clarity, the algorithm exhibiting the best performance for each combination of variable and matrix is underlined.

		Raw	TS	ATS	HP200	ATS + HP200	EKS2	SWT	EMD
64-BP	RE						0.13±0.41		
			0.13±0.04	0.12±0.03	1.30±0.38	1.34±0.34	0.12±0.10	0.47±0.09	0.44±0.08
	SNR						0.80±1.54		
			0.98±0.73	0.95±0.71	0.23±0.19	0.22±0.18	0.73±0.53	0.41±0.29	0.09±0.08
	CC						50.62±15.34		
			54.68±17.33	54.10±17.17	29.91±10.27	29.32±10.05	50.83±15.02	39.53±11.96	19.09±7.01
	SD						583.90±16840.80		
	[0, 20] Hz		3.45±1.74	5.60±2.52	0.01±0.01	0.01±0.01	17.45±19.43	0.07±0.05	1.92±1.51
	SD						113.49±1769.58		
	[20, 200] Hz		77.99±14.31	77.44±14.19	5.84±1.06	5.80±1.06	53.96±9.84	27.23±4.60	7.37±1.84
	KR2						0.62±0.64		
		1.94±1.53	0.07±0.06	0.06±0.05	0.06±0.10	0.06±0.07	0.59±0.32	0.07±0.06	−0.20±0.15
	ΔKR2						−0.25±1.12		
			−0.93±0.08	−0.93±0.08	−0.94±0.09	−0.94±0.08	−0.27±1.03	−0.93±0.09	−0.76±0.53
32-BP	RE						0.14±0.26		0.42±0.07
			0.12±0.03	0.11±0.02	1.19±0.20	1.25±0.18	0.13±0.13	0.44±0.07	0.42±0.07
	SNR						1.02±1.46		0.11±0.15
			1.34±1.08	1.29±1.03	0.29±0.40	0.26±0.35	0.96±0.81	0.53±0.57	0.11±0.15
	CC						55.20±17.98		19.07±10.54
			60.10±20.24	59.48±20.01	30.71±15.38	29.75±14.54	55.26±17.80	41.91±16.06	18.98±10.47
	SD						120.13±1943.55		2.68±3.00
	[0, 20] Hz		4.19±2.44	6.35±2.98	0.02±0.02	0.02±0.02	24.08±31.37	0.09±0.10	2.67±3.01
	SD						60.04±139.65		7.25±3.08
	[20, 200] Hz		78.58±15.41	78.05±15.36	5.67±1.50	5.59±1.45	53.10±12.03	26.68±6.02	7.23±3.08
	KR2						8.29±148.14		−0.06±0.24
		1.99±2.52	0.09±0.09	0.09±0.09	0.09±0.18	0.08±0.09	0.96±0.63	0.09±0.09	−0.05±0.24
	ΔKR2						18.42±349.72		1.69±38.16
			−0.83±0.74	−0.84±0.39	−0.81±1.24	−0.83±1.11	1.18±12.82	−0.86±0.32	1.69±38.25
32-LP	RE						0.27±1.51		0.47±0.13
			0.18±0.06	0.15±0.04	1.31±0.36	1.40±0.30	0.19±0.18	0.55±0.11	0.47±0.13
	SNR						0.39±3.10		0.03±0.06
			0.20±0.36	0.19±0.35	0.07±0.14	0.06±0.13	0.23±0.59	0.12±0.21	0.03±0.06
	CC						24.29±14.79		8.81±7.17
			22.94±15.68	22.77±15.43	13.54±10.63	13.14±10.48	24.45±14.73	18.46±12.50	8.81±7.18
	SD						108,938.36 ± 2,221,637.93		0.81±0.78
	[0, 20] Hz		1.30±0.95	2.27±1.27	0.01±0.01	0.01±0.01	12.81±69.52	0.03±0.03	0.81±0.78
	SD						16,098.75 ± 327,636.46		4.90±2.97
	[20, 200] Hz		42.93±21.43	42.60±21.17	3.46±1.79	3.36±1.83	34.85±41.05	15.88±8.24	4.91±2.97
	KR2						1.17±0.76		−0.01±0.24
		7.50±6.16	0.22±0.18	0.21±0.16	0.15±0.13	0.14±0.12	1.15±0.58	0.15±0.13	−0.01±0.23
	ΔKR2						−0.73±0.27		−0.91±0.21
			−0.95±0.07	−0.95±0.07	−0.96±0.07	−0.96±0.07	−0.73±0.27	−0.96±0.07	−0.91±0.21

**Table 3:** Number of convergence errors observed for each evaluated algorithm: Template Subtraction (TS), Adaptive Template Subtraction (ATS), High-pass filtering with a 200 Hz cutoff frequency (HP200), Adaptive Template Subtraction followed by high-pass filtering (ATS + HP200), Second-order Extended Kalman Smoother (EKS2), Stationary Wavelet Transform (SWT) and Empirical Mode Decomposition (EMD). The results are aggregated across all electrodes and seven subjects, based on two independent trials and are reported for three electrode matrices as well as for the overall dataset. The matrices correspond to the brachial plexus (BP) configuration with 64 electrodes, the BP configuration with 32 electrodes and the lumbar plexus (LP) configuration with 32 electrodes. Each value is expressed as both the absolute number of convergence errors and the corresponding percentage. An additional column reports the total number of cases analyzed for each matrix.

	TS	ATS	HP200	ATS + HP200	EKS2	SWT	EMD	Total
64-BP	0 (0%)	0 (0%)	0 (0%)	0 (0%)	6 (0.67%)	0 (0%)	0 (0%)	896
32-BP	0 (0%)	0 (0%)	0 (0%)	0 (0%)	3 (0.67%)	0 (0%)	2 (0.45%)	448
32-LP	0 (0%)	0 (0%)	0 (0%)	0 (0%)	3 (0.67%)	0 (0%)	2 (0.45%)	448
Total	0 (0%)	0 (0%)	0 (0%)	0 (0%)	12 (0.67%)	0 (0%)	4 (0.22%)	1792

## Data Availability

The original data presented in the study are openly available in Zenodo at [12].

## References

[1] Cadotte D.W., Bosma R.L., Mikulis D.J., Nugaeva N., Smith K., Pokrupa R., Islam O., Stroman P.W., Fehlings M.G. (2012). Plasticity of the Injured Human Spinal Cord: Insights Revealed by Spinal Cord Functional MRI. PLoS ONE.

[2] Valenzuela F., Rana M., Sitaram R., Uribe S., Eblen-Zajjur A. (2021). Non-Invasive Functional Evaluation of the Human Spinal Cord by Assessing the Peri-Spinal Neurovascular Network with Near Infrared Spectroscopy. IEEE Trans. Neural Syst. Rehabil. Eng..

[3] Luger T., Daffertshofer A. Assessing somatosensory evoked potentials using high density surface electromyography grids. Proceedings of the XXI Congress of the International Society of Electrophysiology and Kinesiology.

[4] Redfern M.S., Hughes R.E., Chaffin D.B. (1993). High-pass filtering to remove electrocardiographic interference from torso EMG recordings. Clin. Biomech..

[5] Bartolo A., Roberts C., Dzwonczyk R., Goldman E. (1996). Analysis of diaphragm EMG signals: Comparison of gating vs. subtraction for removal of ECG contamination. J. Appl. Physiol..

[6] Zhan C., Yeung L.F., Yang Z. (2010). A wavelet-based adaptive filter for removing ECG interference in EMGdi signals. J. Electromyogr. Kinesiol..

[7] Lu G., Brittain J.S., Holland P., Yianni J., Green A.L., Stein J.F., Aziz T.Z., Wang S. (2009). Removing ECG noise from surface EMG signals using adaptive filtering. Neurosci. Lett..

[8] Junior J.C., Ferreira D., Nadal J., de Sá A.M. Reducing electrocardiographic artifacts from electromyogram signals with independent component analysis. Proceedings of the 2010 Annual International Conference of the IEEE Engineering in Medicine and Biology.

[9] Andoh T., Ohtsuka T., Okazaki K., Okutsu Y., Okumura F. (1993). Effects of adenosine triphosphate (ATP) on somatosensory evoked potentials in humans anesthetized with isoflurane and nitrous oxide. Acta Anaesthesiol. Scand..

[10] Desmedt J.E. (1985). Critical neuromonitoring at spinal and brainstem levels by somatosensory evoked potentials. Cent. Nerv. Syst. Trauma.

[11] Xu L., Peri E., Vullings R., Rabotti C., Van Dijk J.P., Mischi M. (2020). Comparative Review of the Algorithms for Removal of Electrocardiographic Interference from Trunk Electromyography. Sensors.

[12] Gracia D.I., Iáñez E., Ortiz M., Azorin J.M. (2025). An ESG Database of Spinal Cord Activity During Gait-Related Tasks and Motor Imagery Exercises. Zenodo Repository.

[13] BMIsLab (2025). ReGAIT: Open-source MATLAB ESG Signal Processing Environment. GitHub Repository. https://github.com/bmislab/ReGAIT_ESG/tree/main/ECG_Denoising_Statistics.

[14] Pan J., Tompkins W.J. (1985). A Real-Time QRS Detection Algorithm. IEEE Trans. Biomed. Eng..

[15] Petersen E. (2023). Cardiac Artifact Removal Toolbox. GitHub Repository. https://github.com/e-pet/ecg-removal/releases/tag/1.1.

[16] Petersen E., Sauer J., Grasshoff J., Rostalski P. (2020). Removing cardiac artifacts from single-channel respiratory electromyograms. IEEE Access.

[17] Simoons M.L., Hugenholtz P.G. (1975). Gradual changes of ECG waveform during and after exercise in normal subjects. Circulation.

[18] Donoho D.L. (1995). De-noising by soft-thresholding. IEEE Trans. Inf. Theory.

[19] Mcsharry P., Clifford G., Tarassenko L., Smith L. (2003). Dynamical model for generating synthetic electrocardiogram signals. IEEE Trans. Bio-Med. Eng..

[20] Akhbari M., Shamsollahi M.B., Jutten C., Armoundas A.A., Sayadi O. (2016). ECG denoising and fiducial point extraction using an extended Kalman filtering framework with linear and nonlinear phase observations. Physiol. Meas..

[21] Huang N.E., Shen Z., Long S.R., Wu M.C., Shih H.H., Zheng Q., Yen N., Tung C.C., Liu H.H. (1998). The empirical mode decomposition and the Hilbert spectrum for nonlinear and non-stationary time series analysis. Proc. R. Soc. Lond. Ser. A Math. Phys. Eng. Sci..

[22] Grasshoff J., Petersen E., Rostalski P. Removing strong ECG interference from EMG measurements. Proceedings of the Workshop BIOSIG.

[23] Abbaspour S., Fallah A. (2014). Removing ECG artifact from the surface EMG signal using adaptive subtraction technique. J. Biomed. Phys. Eng..

[24] Thongpanja S., Phinyomark A., Quaine F., Laurillau Y., Limsakul C., Phukpattaranont P. (2016). Probability Density Functions of Stationary Surface EMG Signals in Noisy Environments. IEEE Trans. Instrum. Meas..

[25] Crow E.L., Siddiqui M.M. (1967). Robust Estimation of Location. J. Am. Stat. Assoc..

